# Electrodiagnostic Evaluation of Meralgia Paresthetica

**DOI:** 10.3390/neurosci6030058

**Published:** 2025-06-22

**Authors:** Jernej Avsenik, Simon Podnar

**Affiliations:** 1 Institute of Radiology, University Medical Center Ljubljana, 1525 Ljubljana, Slovenia; jernej.avsenik@kclj.si; 2 Institute of Clinical Neurophysiology, Division of Neurology, University Medical Center Ljubljana, 1525 Ljubljana, Slovenia

**Keywords:** meralgia paresthetica, electrodiagnostics, lateral femoral cutaneous nerve, nerve conduction study, somatosensory evoked potentials

## Abstract

Background: We aimed to determine the utility of different electrodiagnostic (EDx) methods in diagnosing meralgia paresthetica (MP). Methods: Twenty-nine MP patients and 26 controls were included. Sensory nerve action potential (SNAP) and somatosensory evoked potential (SEP) of the lateral femoral cutaneous nerve (LFCN) and tibial SEPs were measured bilaterally. Results: At least one LFCN SNAP was unobtainable in 18 patients (62%) and two controls (8%). In all remaining 11 patients, SNAPs were abnormal at least unilaterally. By contrast, LFCN SEPs were recorded bilaterally in all subjects and were abnormal in 16 patients (sensitivity 48%). Patients’ tibial SEP latency was significantly larger than that of controls (*p* < 0.001). Conclusions: LFCN NCSs are superior to SEP in the evaluation of MP. However, SEP studies may be useful in old (>60 years) and obese subjects with unobtainable LFCN SNAP. Longer tibial SEP points to subclinical neuropathy in MP patients predisposed to LFCN entrapment.

## 1. Introduction

Meralgia paresthetica (MP), the mononeuropathy of the lateral femoral cutaneous nerve (LFCN), is a relatively frequent entrapment neuropathy [1] with an annual incidence rate of 4.3 per 10,000 people [2]. It is usually caused by damage to the LFCN as it passes under the inguinal ligament to enter the thigh medial to the anterior superior iliac spine (ASIS) [3]. Obesity, tight clothing, and pregnancy most commonly predispose to LFCN entrapment below the inguinal ligament. Less common causes include scarring after lower abdominal surgery, tumors, hematomas, and blunt sports injuries of the upper thigh [4]. MP is generally unilateral, although 10% of patients report bilateral symptoms [1].

Patients typically present with varying degrees of numbness, paresthesia, and pain over the lateral aspect of the thigh. Symptoms sometimes worsen with hip extension. Clinically, patients report hypoesthesia or, less commonly, dysesthesia, in the skin area innervated by LFCN [5]. The diagnosis of MP is made clinically. Electrodiagnostic (EDx) evaluation, however, may help to confirm the diagnosis [6,7,8,9] and exclude alternative causes of symptoms [3,7,10]. Multiple mononeuropathies or lumbar plexopathy may be demonstrated by additional nerve conduction studies (NCSs) and by needle electromyographic (EMG) examination [7]. 

MP differs from other compression or entrapment neuropathies in several respects. The LFCN is a pure sensory nerve; therefore, only sensory nerve conduction studies can be considered. It is usually also not possible to stimulate the LFCN proximal to the entrapment site, which reduces the sensitivity of nerve conduction studies. Another difference is that in a typical obese patient with MP, the LFCN lies rather deep below the fat layer and is therefore difficult to stimulate. Similar to median nerve entrapment neuropathy at the wrist, causing carpal tunnel syndrome, meralgia paresthetica can also occur bilaterally, which makes site-to-site comparison less useful. 

Neurophysiological assessment of MP includes NCSs and somatosensory evoked potentials (SEPs) on LFCN stimulation [6]. Abnormalities thought to be consistent with a neurophysiological diagnosis of MP are as follows: (1) undetectable or low amplitude sensory nerve action potential (SNAP); (2) reduced SNAP conduction velocity; (3) delayed or attenuated SEP [7,11]. However, the usefulness of EDx methods is still debated. Some authors consider NCS to be more useful in the diagnosis of MP [12,13] and recommend SEPs only in obese patients [7,12,13]. By contrast, others consider SEP to be the main technique for the objective diagnosis of MP [3,6,14]. In addition, a method based on the latency differences between the tibial nerve (TN) cortical SEPs and LFCN cortical SEPs (parameter D) has been proposed [5].

With the establishment of neuro-muscular ultrasonography use in EDx laboratories, the exact MP diagnosis seems even more relevant today. Surgical release and local steroid injections were found to be similarly effective in the treatment of MP [15]. Therefore, the EDx practitioner has the opportunity to apply ultrasonographic technology both to confirm the diagnosis and to perform controlled LFCN injections [16]. 

The aims of the present study were to (1) assess the reliability and sensitivity of NCSs and SEPs for the evaluation of MP; (2) calculate reference intervals for LFCN NCS and cortical SEP; (3) determine the potential use of tibial SEP in MP; (4) provide technical recommendations for selected measurements; (5) develop an algorithm for the evaluation of patients with clinically suspected MP based on study results; (6) introduce the algorithm into the daily evaluation of patients.

## 2. Materials and Methods

### 2.1. Study Population

A list of patients with a recent clinical diagnosis of uni- or bi-lateral MP was compiled from the database of the Institute of Clinical Neurophysiology, University Medical Centre Ljubljana, Slovenia. Patients were sent a letter inviting them to participate in the study. The control group consisted of the first author’s colleagues and friends. Basic demographic data (age, height, weight) were compiled. All participants’ histories were obtained by a questionnaire, and a neurologic examination was performed. 

### 2.2. Primary Outcome Measures

EDx studies were conducted on a standard EMG system (Nicolet Synergy, Natus Medical, Inc., San Carlos, CA, USA) and included LFCN sensory NCSs, LFCN SEPs, and tibial SEPs. All tests were performed bilaterally in all patients and controls. 

#### 2.2.1. Nerve Conduction Studies (NCSs) 

NCSs were performed using a bipolar surface stimulation electrode (16893; Natus Medical, Inc., San Carlos, CA, USA) and two self-adhesive monopolar surface recording electrodes (019-400400; Natus Medical, Inc., San Carlos, CA, USA). The stimulating anode was placed medially and 1.5 cm distally to the ASIS, with the cathode positioned 2.5 cm distal to the anode. The recording electrodes were located along a straight line between the ASIS and the lateral border of the patella, with the active recording electrode 20 cm distal to the ASIS, and the reference electrode positioned 3 cm more distally (i.e., antidromic measurement). The ground electrode was placed between the stimulating and recording electrodes. Filter settings were 3.2–3200 Hz, a sensitivity 5 μV/division, and sweep speed 1 ms/division. Stimuli were delivered at a frequency of 2 Hz, with a stimulus duration of 0.1 ms. The final SNAP was obtained by averaging 20 sweeps, and two SNAPs were superimposed to ensure reproducibility [7]. Sensory NCSs were defined as abnormal if (1) SNAP was absent; (2) SNAP latency exceeded the upper reference limit (i.e., reduced nerve conduction velocity (NCV)); (3) SNAP amplitude was less than 50% of the opposite side; or (4) side-to-side latency difference exceeded the upper reference limit.

#### 2.2.2. Somatosensory Evoked Potentials (SEPs)

We recorded cortical SEPs from the scalp, using standard surface EEG electrodes (53524T; Natus Medical, Inc., San Carlos, CA, USA) with the active electrode placed 2 cm posterior to Cz and the reference electrode at Fz of the International 10-20 system of electrode placement [7,13].

LFCN SEPs were elicited using two self-adhesive monopolar surface electrodes (019-400400; Natus Medical, Inc., San Carlos, CA, USA) with the stimulating cathode 12 cm below the ASIS and the anode 5 cm more distal on a straight line between the ASIS and the lateral patella border [7]. The ground electrode was placed on the trunk. Filter settings were 3.2–3200 Hz, sensitivity 0.2 μV/division, and sweep speed 10 ms/division. At a stimulation frequency of 2 Hz, 200 stimuli (duration 0.2 ms) were delivered. Two recordings were superimposed, and the latency of the first positive peak (P30) was measured [7]. We defined SEP abnormality as follows: (1) absent P30 wave; (2) P30 latency exceeding the upper reference limit; or (3) a side-to-side latency difference exceeding the upper reference limit.

For tibial SEPs, the TN was stimulated at the ankle, using two self-adhesive monopolar surface electrodes (019-400400; Natus Medical, Inc., San Carlos, CA, USA). Filter settings were 3.2–3200 Hz, sensitivity 0.2 μV/division, and sweep speed 10 ms/division. For each response, 100 stimuli of 0.2 ms duration were delivered at 5 Hz. Two recordings were superimposed, and the latency of the first positive peak (P40) was measured. The latency differences between cortical tibial SEPs and LFCN SEPs (parameter D) were calculated. We defined abnormality as follows: (1) a D value below the lower confidence limit, or (2) a side-to-side D difference exceeding the upper confidence limit.

### 2.3. Statistical Analysis

All reference limits were calculated in the control group using parametric statistics (mean ± 2 SD). Statistical comparisons were made using the Student’s *t*-test. We calculated each test’s sensitivity, specificity, positive predictive value (PPV), and negative predictive value (NPV) using the conventional 2 × 2 table. The Chi-square test was used to determine the association between NCSs and SEPs in their ability to record a response. Based on our data and previously reported studies [7,12,13], an algorithm for the EDx evaluation of patients with MP was formulated. The sensitivity and specificity of the protocol on our data were calculated.

### 2.4. Ethic Statements

The study was approved by the National Ethics Committee of Slovenia, and all subjects provided informed consent before their inclusion into the study.

## 3. Results

A total of 29 patients (15 men), and 26 healthy controls (15 men) were included in the study. Unilateral MP was clinically diagnosed in 25 patients, and bilateral MP in four patients. The patient group was significantly older (median age: 59 years vs. 26 years; range: 20–78 years vs. 21–61 years; *p* < 0.001), shorter (median height: 168 cm vs. 177 cm; range: 145–193 cm vs. 162–194 cm; *p* < 0.001), and heavier than the control group (median body mass index (BMI): 28.4 kg/m^2^ vs. 22.2 kg/m^2^; (*p* < 0.001)). 

### 3.1. History and Clinical Neurologic Examination

Numbness over the lateral aspect of the thigh was reported by 21 patients (72%), paresthesia by 18 patients (62%), and burning pain by 16 patients (55%). On neurologic examination, 21 patients reported hypesthesia (72%), 17 patients hypalgesia (59%), four patients hyperalgesia (14%), and four patients dysesthesia (14%). A positive Tinel’s sign, elicited by tapping medial to the ASIS, was reported by 12 patients (41%). In control subjects, no abnormalities were found on neurologic examination. 

### 3.2. Criteria for Neurophysiological Diagnosis of MP

Based on the results in our control subjects, we calculated the criteria for the EDx diagnosis of MP. For LFCN NCSs, the following criteria were obtained: (1) unrecordable LFCN SNAP; (2) LFCN conduction velocity <56.4 m/s; (3) LFCN SNAP amplitude <50% compared to the opposite side; (4) side-to-side conduction velocity difference >6.4 m/s. For LFCN SEP, the following criteria were obtained: (1) unrecordable LFCN SEP; (2) P30 latency >35.6 ms; (3) side-to-side latency difference > 5 ms.

### 3.3. Nerve Conduction Studies (NCSs)

**Figure 1:**
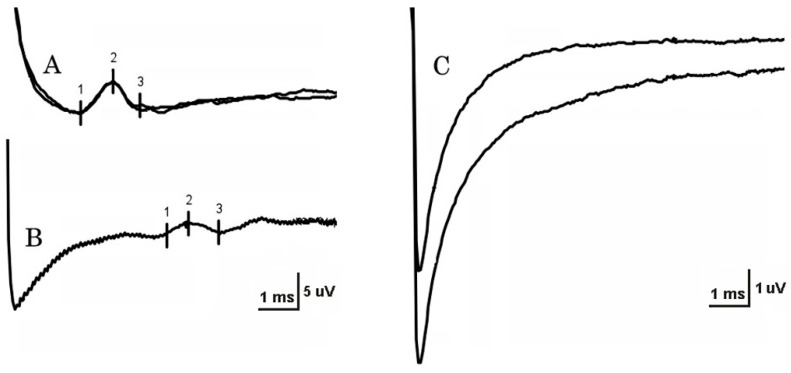
Nerve conduction studies (NCSs) of lateral femoral cutaneous nerves (LFCNs). (**A**) Normal LFCN NCS results in a healthy 25-year-old woman with a BMI of 20 kg/m^2^. (**B**) Abnormal NCSs in a 54-year-old overweight man (BMI = 34 kg/m^2^) with bilateral meralgia paresthetica (MP). Note increased latency and reduced amplitude sensory nerve action potential (SNAP) of the left LFCN. (**C**) Non-recordable right LFCN SNAP in the same patient as in B.

Stimulation strength varied from 4 to 20 mA on NCSs. At least one LFCN SNAP was unobtainable in 18 patients (62%) and in two controls (8%) (Figure 1), a 28-year-old woman (BMI 24) and a 61-year-old man (BMI 36). On the unaffected side, reproducible SNAPs were recorded in 13 of 25 patients (52%) with unilateral MP. In patients, LFCN SNAP conduction velocity was significantly reduced on the affected side in comparison with both the unaffected side and the control group (*p* < 0.001). SNAP amplitude in patients was significantly lower on the affected side in comparison with the control group, but not in comparison with the unaffected side (Table 1). The side-to-side difference between conduction velocity and SNAP amplitude was significantly larger in the group of patients with unilateral MP in comparison with the control group (Table 2). The most frequent abnormality of NCSs in patients was increased side-to-side conduction velocity difference (32%), followed by reduced conduction velocity (30%).

### 3.4. Somatosensory Evoked Potentials (SEPs)

**Figure 2:**
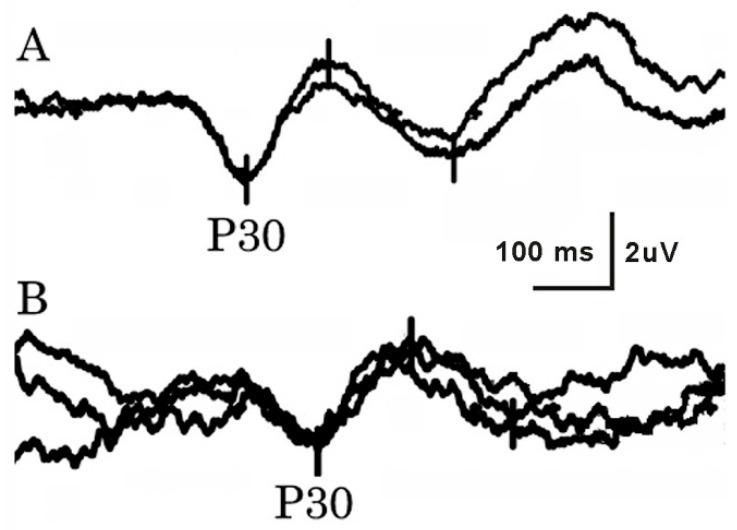
Somatosensory evoked potential (SEP) on stimulation of the skin innervated by the lateral femoral cutaneous nerve (LFCN). (**A**) Normal LFCN SEP in a healthy 25-year-old woman (BMI 25 kg/m^2^). (**B**) Abnormal LFCN SEP in a 66-year-old woman (BMI 34 kg/m^2^) with bilateral meralgia paresthetica (MP). Note the increased latency of the first positive peak (P30).

Stimulus strengths varied from 6.5 to 20 mA for LFCN, and from 6.5 to 40 mA for tibial SEPs. Both LFCN and tibial SEPs were bilaterally obtained in all tested subjects (Figure 2). NCSs and SEPs differed significantly (*p* < 0.001) regarding their ability to record a response. P30 latency after LFCN stimulation was significantly increased on patients’ affected sides in comparison with both patients’ unaffected sides and the control group. The latency difference between the cortical tibial SEP and LFCN SEP (parameter D) was significantly lower on the affected side in comparison with the unaffected side, but not in comparison with the control group (Table 1). The side-to-side difference of P30 latency and parameter D was significantly higher in the group of patients with unilateral MP in comparison with the control group (Table 2). The most frequent LFCN SEP study abnormality in MP patients was increased P30 latency (42%), followed by increased side-to-side latency difference (28%). Parameter D was below the threshold in 36% of patients. The tibial SEP latency in patients was significantly larger compared to controls (Table 1).

### 3.5. Sensitivity, Specificity, Positive (PPV), and Negative Predictive Value (NPV)

NCS was abnormal in all 11 patients (38%), with a response obtained at least unilaterally (sensitivity 100%, specificity 85%, PPV 78%, NPV 100%). LCFN SEP was abnormal in 16 patients (sensitivity 48%, NPV 58%), while parameter D was abnormal in nine studied patients (sensitivity 27%, NPV 50%). The sensitivity and specificity of the suggested EDx algorithm for the EDx evaluation of patients with MP in our population were 88% and 84%, respectively.

## 4. Discussion

**Figure 3:**
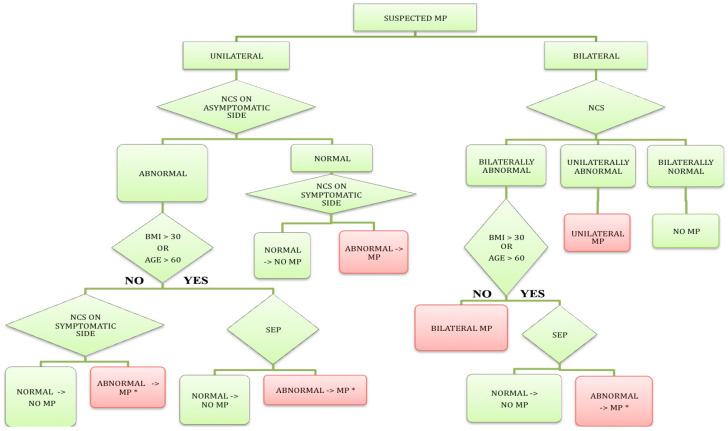
Diagnostic algorithm for electrodiagnostic evaluation of patients with suspected meralgia paresthetica (MP) based on findings of the present and previous studies [7,12,13]. Legend: NCS—nerve conduction study of the lateral femoral cutaneous nerve (LFCN); SEP—somatosensory evoked potentials on LFCN stimulation; BMI—body mass index [kg/m^2^]; *—to exclude generalized pathology, additional peripheral NCS and/or tibial SEPs are recommended.

Our study demonstrated LFCN NCSs to be superior to LFCN SEP in the neurophysiological evaluation of MP. NCS results were abnormal in all patients in whom a response was obtained at least unilaterally (sensitivity 100%), while SEP results were abnormal in 48% of patients. On the other hand, LFCN SNAP was at least unilaterally unobtainable in two control subjects (8%) and in 18 patients (62%), while LFCN SEPs were recorded bilaterally in all patients and controls. Parameter D was abnormal in only 27% of patients, suggesting that tibial SEPs are not helpful in the diagnosis of MP. Of the two controls with unobtainable LFCN SNAP, one was young (< 60 years) and thin (BMI < 30 kg/m^2^), and the other was old (>60 years) and overweight (BMI > 30 kg/m^2^). As there was only one control subject older than 60 years or with BMI over 30 kg/m2, these subjects represented 4% and 100% of our control population with age < 60 years and BMI < 30 kg/m^2^, and age > 60 years or BMI >30 kg/m^2^, respectively. These data are therefore in accordance with previous studies reporting unobtainable LFCN SNAP in a high proportion of controls with age > 60 years or BMI > 30 kg/m^2^ [7,12,13]. Based on these data, we proposed a diagnostic algorithm for the EDx evaluation of patients with suspected MP (Figure 3).

Our reference intervals are similar to those obtained in previous studies using needle electrodes [7,12]. However, differences in the applied technique (i.e., surface vs. needle electrodes) probably explain why the most frequent neurophysiological abnormality in our study was reduced SNAP conduction velocity, compared to low SNAP amplitude in previous studies [7,17]. Results similar to ours have been previously reported by Spevak and Prevec, who also used surface electrodes [18]. Our SNAP conduction velocities were similar to those reported previously [7,11,12,13,18,19]. Our LFCN SNAP measurements were performed distal to the entrapment site. Reduced conduction velocity is expected to occur due to changes in sensory nerve axons and possibly also in myelin in the distal segment.

The high percentage of patients with unobtainable LFCN SNAP on the unaffected side in our study may indicate subclinical LFCN entrapment. It has been estimated that LFCN NCS becomes unrecordable using surface electrodes when 90% of nerve axons are lost [13]. The low amplitude or unobtainable LFCN SNAP is caused primarily by axonal degeneration distal to the entrapment site. Moreover, the high BMI in many of these patients may also contribute to the difficulty in obtaining SNAPs without needle electrodes [17]. Using surface electrodes, it is more difficult to detect low-amplitude SNAPs in overweight people with a thick subcutaneous fat tissue layer. In addition, a previous study suggested that age over 60 years also affects the NCS results [13]. It is well known that aging reduces the number of axons in peripheral nerves. Another explanation is the high variability of the anatomical course of LFCN [10,18,20]. Therefore, it is more difficult to find the exact course of the nerve. This is important due to the sharp reduction in SNAP amplitude with inaccurate positioning of the recording electrodes. This, however, is expected to affect the needle electrode technique even more. The lower amplitude of SNAPs on the affected side in our study compared to previous reports [7,17] may be due to the long disease duration (mean, 4 years) in our patients. 

On the other hand, the high sensitivity of LFCN SEP has been reported by Esteban [6] and el-Tantawi [14]. On their way to the somatosensory cortex, SEPs are amplified, which secures their presence, even in severely affected patients. Furthermore, on elicitation of LFCN SEP, SNAPs pass the entrapment site. However, the entrapment segment and the segment with reduced conduction velocity distal to the entrapment are “diluted” in the long unaffected peripheral and central pathway proximal to the entrapment site. Esteban recorded cortical potentials both medially and contralaterally to the stimulation, supporting the suggestion that multiple LFCN SEP recording montages on the scalp would be useful [6]. In addition, Caramelli et al. proposed a calculation of the latency difference between cortical tibial SEP and the cortical LFCN SEP (parameter D) [5]. Theoretically, this approach would be useful because it reduces intrinsic variability between subjects. In our study, however, we failed to confirm the high sensitivity and specificity of this parameter. P30 latencies in our patients were much shorter than in the study of Caramelli et al. (mean, 35.4 ms vs. 40.1 ms, respectively) [5]. This might be due to more severe MP in their population. The larger tibial SEP latency in our patients compared to controls is also interesting (Table 1). This remained significant (*p* < 0.001) even after eliminating nine patients with diabetes mellitus and/or possible lumbosacral radiculopathy. Although the finding could be partly explained by the higher patients’ age, interestingly, controls were significantly taller than patients, which would be expected to more than compensate for the age difference [21,22,23]. The tibial SEP latencies in our patients were also larger compared to previously reported reference values adjusted for the control subjects’ age and height [21,23]. Our observation may therefore point to an underlying generalized sensory pathway involvement in the population of MP patients. Such involvement might predispose these individuals to MP. This finding emphasizes the need for additional peripheral NCS in some patients with clinically suspected MP (Figure 3).

As previously suggested [7,12,13], our results support the possible role of LFCN SEP measurement in obese (BMI > 30 kg/m^2^) and old (>60 years) patients with suspected MP. We believe LFCN SEP is particularly useful in patients in whom LFCN SNAPs are unobtainable on the asymptomatic side. If SNAP is absent on the asymptomatic side, it is impossible to interpret an unobtainable SNAP on the symptomatic side as pathologic [14].

### 4.1. Diagnostic Algorithm for EDx Evaluation of MP

Given the high sensitivity of LFCN NCSs on the one hand, and the ability of SEP to record a response from all patients and controls on the other hand, we constructed a diagnostic algorithm for the EDx evaluation of patients with suspected MP (Figure 3). The algorithm is, furthermore, based on previous studies [7,12,13] demonstrating unobtainable LFCN SNAP, but not LFCN SEP in a large proportion of older (>60 years) or obese (BMI > 30 kg/m^2^) controls. 

In patients with unilateral MP, abnormal NCSs on the asymptomatic side are, in our opinion, a sign of subclinical LFCN disorder. If the patient is young with a BMI < 30 kg/m^2^, we believe that the result of NCSs on the symptomatic side either confirms or excludes the diagnosis. By contrast, in older (>60 years) and/or obese patients (BMI > 30 kg/m^2^) [12] with abnormal NCSs on the asymptomatic side, an LFCN SEP study is needed to confirm or exclude the diagnosis on the symptomatic side. Furthermore, in subjects with normal NCSs on the asymptomatic side, the diagnosis is confirmed or excluded by the NCS on the symptomatic side alone. 

In young (<60 years) and non-obese (BMI < 30 kg/m^2^) patients with clinically suspected bilateral MP, only LFCN NCSs are needed for neurophysiological confirmation of the diagnosis. By contrast, in old (>60 years) and/or obese (BMI > 30 kg/m^2^) patients with bilaterally abnormal NCS, LFCN SEP also must be performed to confirm or exclude MP. We demonstrated good sensitivity (88%) and specificity (84%) of the suggested EDx algorithm in our population.

### 4.2. Study Limitations

The study was performed in relatively small groups of patients and controls. The patient group was significantly older (*p* < 0.001) and heavier (*p* < 0.001) than the control group. These differences might result in the more stringent reference intervals obtained in our control population, leading to increased sensitivity and reduced specificity of EDx studies. However, it is unknown whether these differences would affect the relative utility of LFCN NCSs and LFCN SEPs in diagnosing MP obtained in the present study. 

## 5. Conclusions

In the evaluation of patients with MP, LFCN NCSs are superior to SEP. However, in old (>60 years) and obese subjects with unobtainable LFCN SNAP, SEP studies may be useful. In MP patients predisposed to LFCN entrapment, longer tibial SEP latencies point to subclinical neuropathy. 

## Figures and Tables

**Table 1:** Results of nerve conduction studies (NCSs) and somatosensory evoked potentials (SEPs) in 29 patients with meralgia paresthetica (MP) and 26 healthy controls.

	Patients		Controls		*p*-Values	
	Symp Side	Asymp Side		Symp vs. Asymp	Symp vs. Controls	Asymp vs. Controls
NCSs						
Amplitude [μV]	0.95 ± 1.5	1.7 ± 2.3	3.2 ± 1.5	0.14	<0.001	0.007
Conduction velocity [m/s]	48.7 ± 8.3	60.1 ± 3.7	60.8 ± 2.2	<0.001	<0.001	0.49
SEPs						
P30LFCN latency [ms]	35.4 ± 3.3	31.2 ± 2.0	31.5 ± 2.0	<0.001	<0.001	0.49
P40TN latency [ms]	45.6 ± 5.8	44.2 ± 3.5	40.6 ± 4.3	0.27	<0.001	0.002
D latency [ms]	10.3 ± 6.1	13.0 ± 3.6	9.0 ± 4.2	0.05	0.38	<0.001

Asymp—asymptomatic; D latency—latency difference between the tibial and lateral femoral cutaneous nerve SEP; LFCN—lateral femoral cutaneous nerve; Symp—symptomatic; TN—tibial nerve.

**Table 2:** The side-to-side difference in electrodiagnostic findings in 25 patients with unilateral meralgia paresthetica (MP) and 26 healthy controls.

	Patients	Controls	*p*-Value
Lateral femoral cutaneous nerve NCSs			
Conduction velocity [m/s]	13.0 ± 7.7	2.8 ± 1.8	<0.001
Amplitude [μV ]	2.1 ± 0.9	1.2 ± 1.3	0.038
Somatosensory evoked potentials (SEPs)			
P30LFCN latency [ms]	3.8 ± 2.7	1.6 ± 1.7	0.001
D latency [ms]	3.9 ± 3.5	2.0 ± 1.9	0.019

D latency—latency difference between the tibial and lateral femoral cutaneous nerve SEP; LFCN—lateral femoral cutaneous nerve; NCS—nerve conduction studies.

## Data Availability

The authors will make the raw data supporting this article’s conclusions available upon request.

## Abbreviations

The following abbreviations are used in this manuscript:

ASIS	The anterior superior iliac spine
BMI	Body mass index
EDx	Electrodiagnostic
EMG	Electromyographic
LFCN	The lateral femoral cutaneous nerve
MP	Meralgia paresthetica
NCSs	Nerve conduction studies
NCV	Nerve conduction velocity
NPV	Negative predictive value
P30	The first positive peak of LFCN SEPs
P40	The first positive peak of tibial SEPs
PPV	Positive predictive value
SEP	Somatosensory evoked potential
SNAP	Sensory nerve action potential
TN	Tibial nerve
